# Use of thermography in skin grafts after the application of therapeutic ultrasound in Wistar rats^1^

**DOI:** 10.1590/s0102-865020200070000003

**Published:** 2020-08-05

**Authors:** Ynaiê Garcia da Silveira, Caroline Kajiura, Filippo Jannoni Lessa Bernardes, Sônia Prince Maria, Marina Pugnaghi Fernandes, Marla Tereza Frasson, Pedro Cassino, Stella Habib Moreira, Jorge Luis Álvarez Gómez, Josiane Morais Pazzini, Andrigo Barboza de Nardi, Paola Castro Moraes

**Affiliations:** I Graduate student, Faculdade de Ciências Agrárias e Veterinárias, Universidade Estadual Paulista (FCAV-UNESP), Jaboticabal-SP, Brazil. Conception and design of the study; acquisition, analysis and interpretation of data; manuscript preparation.; II Graduate student, FCAV-UNESP, Jaboticabal-SP, Brazil. Acquisition of data.; III Graduate student, FCAV-UNESP, Jaboticabal-SP, Brazil. Acquisition, analysis and interpretation of data.; IV Veterinarian, FCAV-UNESP, Jaboticabal-SP, Brazil. Acquisition of data.; V Veterinarian, FCAV-UNESP, Jaboticabal-SP, Brazil. Manuscript preparation and writing.; VI Fellow Master degree, Postgraduate Program in Veterinary Surgery, FCAV-UNESP, Jaboticabal-SP, Brazil. Acquisition of data, technical procedures.; VII Fellow PhD degree, Postgraduate Program in Veterinary Surgery, FCAV-UNESP, Jaboticabal-SP, Brazil. Conception and design of the study; acquisition, analysis and interpretation of data; manuscript preparation.; VIII Fellow PhD degree, Postgraduate Program in Veterinary Surgery, FCAV-UNESP, Jaboticabal-SP, Brazil. Acquisition, analysis and interpretation of data; technical procedures; histopathological examinations; manuscript preparation and writing; critical revision.; IX Fellow PhD degree, Postgraduate Program in Veterinary Surgery, UNESP/FCAV, Jaboticabal-SP, Brazil. Acquisition, analysis and interpretation of data; technical procedures; manuscript preparation and manuscript writing.; X Professor, União das Faculdades dos Grandes Lagos (UNILAGO), Sao Jose do Rio Preto-SP, Brazil. Conception and design of the study, analysis and interpretation of data, technical procedures, histopathological examinations, statistics analysis, manuscript preparation and writing, critical revision, final approval.; XI Full Professor, Department of Clinical and Veterinary Surgery, FCAV-UNESP, Jaboticabal-SP, Brazil. Conception and design of the study.; XII Full Professor, Department of Clinical and Veterinary Surgery, FCAV-UNESP, Jaboticabal-SP, Brazil. Conception and design of the study, technical procedures, manuscript preparation and writing, critical revision, final

**Keywords:** Thermography, Skin, Rat

## Abstract

**Purpose:**

To analyze changes in the thermal pattern in the skin graft receptor bed, after the use of therapeutic ultrasound through the thermographic images.

**Methods:**

Eighteen Rattus norvegicus albinus Wistar, separated into two groups: GST groups (without tumor and without treatment with ultrasound) and GT (with tumor and treatment with ultrasound). In the GT group, induction of carcinogenesis was performed by single intradermal application of 0.05 ml DMBA at 0.5%, diluted in acetone. Subsequently, a technique of reconstructive grafting surgery of the mesh type was performed in both groups and treatment with therapeutic ultrasound was performed in the GT group the alternate day protocol at 3, 6, 10 and 15 days after the procedure. The thermographic evaluation occurred on days 3, 6, 10 and 15 after the grafting.

**Results:**

There was a significant difference between the statistical evaluation of the temperature of the control group when compared to the treated group, on the different evaluation days (p <0.0001).

**Conclusion:**

The thermographic analysis of the images was effective in evaluating the healing process, being the use of thermography feasible to evaluate changes in the thermal standard in the surgical bed, besides the beneficial effects of the US.

## Introduction

Assuming the physical consideration that every living organism produces heat and thereby emits infrared radiation directly proportional to its temperature, thermography consists of the immediate and non-aggressive evaluation of the mapping of this radiation charge, expressing the gradual gradient variation in color pattern in a thermogram^1^. According to Corte and Hernandez^2^, when detecting infrared radiation, innumerable alterations related to changes in blood flow can be identified, being possible the recognition of vascular, neurological and muscular functional alterations.

To obtain thermal images, it is necessary to perform data analysis of the thermogram software, which allows the reading of images, inspection, obtaining results and the capture of changes from 0.05°C^3^. Some factors must be taken into account in order to obtain accurate thermographic analyses, such as environmental factors as temperature, relative air humidity and atmospheric pressure, technical elements as protocols, software and statistical analysis, and individual aspects of animals as weight, age and circadian rhythm^4^.

Reconstructive surgery is a branch that has gained prominence within this context due to its importance in the practice of tissue reconstruction involving the use of cutaneous grafts. The grafts refer to the insertion of a fragment of the dermis and epidermis at a pre-established distant receptor site^5^. According to Sun *et al*.^6^, several techniques for implantation can be performed; however, precautions with the use of accurate technique should be established, reducing complications and ensuring adequate post-surgery.

At each step of the skin recovery, the cicatricial process is of fundamental importance for the restoration of the contiguity of the skin. In addition, the formation and expansion of new vessels, called angiogenesis, also plays an essential role in the recovery process, since it promotes irrigation in sites that previously did not have vascularization^6^.

Within the clinical and surgical medical routine, numerous practices assist in promoting the functional recovery of tissues. One of the procedures that has gained prominence today is the therapeutic ultrasound (UST), used in human medicine^7^. According to Farcic *et al*.^8^, ultrasound is a category of sonic energy of high penetrability in the tissues that during the contact has the ability to originate cellular transformations due to the absorption of the mechanical energy. As a consequence of the presence of a crystal inside the transducer, the mechanical energy is produced through the transmission of electric current, being denominated of piezo electric effect.

For therapeutic ultrasound to present a biologically ambitious thermal impact, it should be applied for approximately five minutes and reach a temperature between 40°C and 45°C in the target tissue^9^. Due to the fact that the technique has the directed heat resource, in other words, the UST is applied in a certain area, penetrates the tissue, one part of the reflected energy and the other absorbed generating heat, having greater benefits over other commonly used procedures^10^.

With the elevation of the temperature promoted by the UST, beneficial effects of pain attenuation, also of the acute or chronic inflammatory process, blockage of muscle contractions/spasms and increase of the extensibility of collagen can be observed^9,11^. Highly appreciated non-thermal effects can also be observed, such as increased vascular permeability, protein production, calcium ion flux and also metabolite transit through the cell membrane, contributing significantly to the improvement of the repair capacity of tissues^12^.

Furthermore, when referring to cancer tumor angiogenesis, which has been shown to be a potential biological marker of behavior and prognosis, and the use of regenerative therapies, such as ultrasound, laser therapy and other methods, are discussed to be used after surgical resection of tumors, because the behavior of the lesions is not known, especially if they can be influenced by ultrasonic waves.

Therefore, the use of thermography in these cases is a tool to assist in the assessment of repair after a surgical procedure, as well as in the diagnosis of neovascularization in the surgical bed after the use of regenerative therapies.

The objective of this study was to evaluate the thermographic image, changes in the thermal pattern in areas where the skin graft was performed in rat after surgical resection of chemically induced squamous cell carcinoma and to evaluate if thermographic could help in locating the identification of angiogenesis that favors the stimulation of the repair of surgical wounds.

## Methods

This study was approved by the Committee on Ethics in the Use of Animals (CEUA), Universidade Estadual Paulista (UNESP), *campus* Jaboticabal (Protocol number 00997917).

### *Experimental animals and groups*

The procedures of this study were performed at the “Governador Laudo Natel” Veterinary Hospital, Faculdade de Ciências Agrárias e Veterinárias, Universidade Estadual Paulista (FCAV-UNESP).

The experimental model used to perform the reconstructive surgery was the rat *Rattus norvegicus albinus* Wistar. 18 healthy male rats with an average age of 21 days and weighing around 30g from the Central Biotherm of the Faculty of Veterinary Medicine, UNESP, *campus* Botucatu, were used. The rats were kept in plastic cages, housed in a climate-controlled environment, in the animal facilities of the Veterinary Surgery Postgraduate course, under environmental conditions at a temperature of 20° and controlled humidity, and were fed with balanced commercial feed and free water, as recommended by the National Council for the Control of Animal Experimentation (CONCEA).

The animals were separated into two groups of 9 animals: GST group (without tumor and without treatment with therapeutic ultrasound) and GT (with tumor and treatment with therapeutic ultrasound).

### *Induction of neoplasia*

Carcinogenesis induction was performed by single intradermal application of 0.5% DMBA (7,12-dimethylbenzanthracene, Sigma-Aldrich, USA) diluted in acetone to the skin of the dorsal region of the thorax, 3 cm in the caudal direction to the first cervical vertebra, as described by Oliveira *et al*.^13^. Due to the safety of the team, the procedure was performed in a laminar flow chamber, with the use of individual protection (PPE).

In the first week of the experiment, the solution was applied^14^, and the animals were evaluated for a period of 16 weeks to analyze the progression of carcinogenesis induction^15^, assessing macroscopic changes due to the process, such as skin texture, nodule growth, tumor diameter, staining, as well as the percentage of animals that developed the tumor macroscopically. The lesions reached a maximum size of approximately 0.05 mm.

### *Surgical procedure*

**Figure 1:**
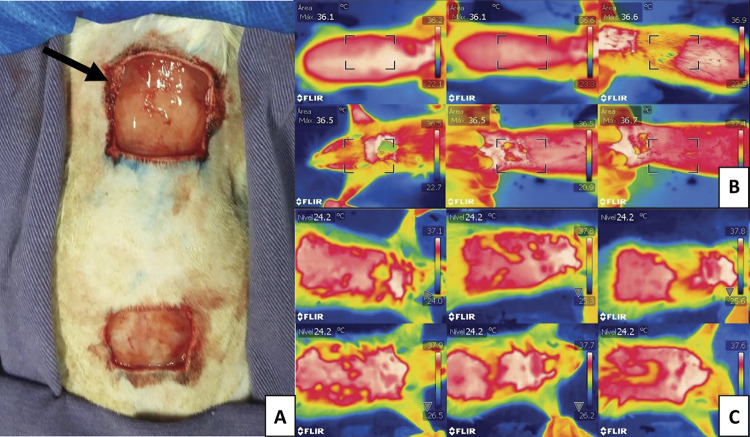
Photographic image of the grafting surgical procedure performed in a rat. A. Images of reconstructive surgery procedures in rats (*Rattus norvegicus albinus* Wistar), arrow indicating the graft recipient bed and caudal region the graft donor bed “Governador Laudo Natel”, FCAV-UNESP, 2018. B. GC Group on the 15th day. C. GT Group on the 15th day.

The same grafting reconstructive surgery technique was performed in the animals. The groups differed only in the treatment with therapeutic ultrasound after the surgical procedure. The surgical procedure was started with an extensive trichotomy of the thorax dorsal face and an antisepsis with chlorhexidine and 90% alcohol solution in the donor region, as well as the receiving region. The anesthetic induction and maintenance were performed with 1 to 3% of isofluorane diluted in 100% of oxygen, using an anesthetic vaporizer. For the neoplastic lesion demarcation in the thorax dorsal portion, a surgical pen and sterile ruler were used. With a No 15 scalpel blade, a 1cm^2^ lesion in a square format was exceeded. Then, the skin graft was obtained in the thorax dorsal region. A cutaneous incision was made in a square format, measuring 1 cm^2^, advancing 6 cm caudally to the spinous process of the first vertebra, being important that the size of the graft was equal to the defect created in the receiving region. After the skin incision, the graft was carefully dissected and removed from the donor region, ending this step with the removal of fat residues and the subcutaneous tissue from the fragment, as well as the slits confection in the graft. The sutures in a separate simple pattern, using 4-0 nylon, were first distributed at the square vertices between the tissue of the donor region with the receiving one, always in the graft direction to the skin of the receiving region, in order to avoid graft movement at the suture time, and then, the rest of the surgical wound was synthesized. The donor region wound in the right thorax was submitted to dermorraphy in a geometric pattern shape, with 4-0 nylon. The sutures starter at the defect vertices and converged towards the center in the surgical wound, at the end they presented a surgical scar with two inverted triangles. All animals received meperidine at a dose of 20mg/kg/subcutaneously, every 12 hours, for 7 days (Fig. 1).

### *Use of therapeutic ultrasound*

The animals of the GT group, submitted to the surgical procedure, were observed for a period of 15 days, performing evaluations in relation to the healing aspect through the application of ultrasound. The application was performed with a probe of 4 cm, frequency of 3Mhz and intensity of 0.1 W/cm^2^, following the protocol of alternate days of application, done by a blind observer at 3, 6, 10 and 15 days post- surgery.

### *Skin temperature evaluation*

The evaluation of the thermographic images of the skin grafts was done regularly in the same period; moreover, there was no influence of the circadian rhythm of the animals. For he GST group, the evaluation was performed on days 3, 6, 10 and 15 postoperatively. In the GT group on days 3, 6, 10 and 15. To perform the evaluation, the FLIR^®^ T-300 camera was used, adjusted for focusing at 30cm distance with automatic focus regulation, captured temperature range ranging from -20 to 650 degrees Celsius, thermal sensitivity of 0.05 degrees and IR resolution of 320 x 240 (76800 pixels). According to Ferreira *et al*.^16^, the thermal mapping, which allows the interpretation of the images, was performed by software, responsible for the investigation and measurement of the absolute, maximum, mean and minimum temperature of the region of interest, defined by the creation of spots.

### *Statistical analysis*

For the analysis, software R (RTM Foundation for Statistical Computing, Vienna, Austria) was used. Initially, the hyposedasticity of the variances (Barlett’s test) and the residue normality (Shapiro’s test) were tested. The actual or transformed measurements of the temperature obtained by the thermography technique were compared between the treatments, moments and their interaction using the ANOVA test with repeated measures in time, a significance set at 5% (P <0.05) and the data presented as the mean ± standard deviation (SD).

## Results

There was a significant difference between the control group when compared to the treated group on the different evaluation days. In the treated group, there was a significant difference on days 5, 10 and 15 (p <0.0001), and it was possible to observe that the temperature raised on the fifth day and remained stable until the end of the evaluations. In the control group, the temperature decreased at the beginning of the healing phase (p <0.0001), between days 5 and 10, to recover on day 15, whereas in the GT group it was significantly higher (p <0.0001) on days 5, 10 and 15 than on day 3 (Table 1). However, the average temperature, minimum and maximum, showed the same behavior (p = 0.7890) when compared between groups.

**Table 1:** Statistical evaluation of the average temperature between the treated and control groups over the analysis days. “Governador Laudo Natel”, FCAV-UNESP, campus Jaboticabal, 2018.

Days after grafting	Average Temperature control group (°C)	Average Temperature treated group (°C)	p value
3	*34.3*	*35*	*p>0.05*
5	*33.8*	*35.5*	*p<0.0001*
10	*33.3*	*35.6*	*p<0.0001*
15	*34.3*	*36.0*	*p<0.0001*

The results showed that tissue repair in the treated group was better when compared to the control group (p <0.05), and no signs of tumor recurrence were observed through the use of therapeutic ultrasound during the 15-day postoperative period.

## Discussion

According to Oliveira *et al*.^17^, the effect of temperature on healing is directly related to its effect on peripheral vasomotor tone, exerting autonomic reflex vasodilation, which promotes increased local vasodilation and oxygenation and tissue nutrition, indicating the formation of new vessels during the process scarring. Therefore, it can be inferred that healing was favorable in the animals treated with therapeutic ultrasound, since the increase of the temperature promotes the expansion of new vessels, denominated angiogenesis, not being observed in the control group that did not receive therapeutic ultrasound.

According to Maia Filho *et al*.^18^, other effects of the use of US constitute the rearrangement and increase of the extensibility of the collagen fibers, improvement of the mechanical properties of the tissues and increase of the circulation. In the present study, the heat production by the use of the US in the treated group led to thermal alteration in the tissue, promoting vasodilation and, consequently, increased regeneration and vascularization, when compared to the control group.

The results of this study are similar to those performed by Altomare *et al*.^19^, who analyzed the effects of US on the process of wound healing by ischemia in Wistar rats, verifying greater and better wound contraction on days 7 and 14 in the treated group when compared to the control group. In this present study, the US used in the animals of the treated group was also effective in the tissue repair, since the temperature remained constant throughout the development, indicating an ideal healing process.

Studies indicate that the growth and metastasis of various tumors can occur due to the transformation from a pre-vascular phase to an angiogenic phase^20^. In addition, angiogenesis is likely to be the result of an imbalance between negative and positive stimuli, such as paracrine mechanisms and local angiogenic factors^21^. However, this process does not occur immediately, it takes time for all of these changes to be installed. This fact may suggest that, in this study, at 15 days of analysis, no presence of angiogenesis was detected in the repair process that could be related to tumor recurrence, but it was through the use of therapeutic ultrasound, which was beneficial for the healing of the surgical wound.

In this study, a better tissue repair was observed in the group treated with therapeutic ultrasound when compared to the control group, the issue of tumor resection does not interfere with the healing process, but it is mainly related to the issue of recurrent tumor lesions. There was no tumor recurrence lesion in the 15-day period that could compromise the use of therapeutic ultrasound, but this time is too short to state that this treatment modality does not cause any risk to cancer patients. In this study, it can be inferred that the use of therapeutic ultrasound is indicated for the repair of skin wounds, and in addition, the use of thermography as an alternative to evaluate the evolution of this tissue repair process by measuring the temperature of the wound bed. Thus, the results found related to tissue repair favorable to the use of therapeutic ultrasound are consistent with the literature^22^.

The chemically induced tumors in the animals were used to assess whether, using the therapeutic ultrasound during the postoperative period, neoplasm recurrence would be detected. However, as the experiment was short, this information could not be analyzed in these groups, but in another group, which does not belong to this work, this information was analyzed and it will be published later.

Regarding the healing of the surgical wound, when the procedure is performed with wide margins, as was done in this study and the tumor does not present invasion in the wound bed, a high healing success rate, without interference in this process. The results obtained in these studies are in agreement with Pazzini *et al*.^23^ These authors suggest wide margins for surgical resection of neoplasms and the use of reconstructive surgery techniques to obtain successful treatment without interfering with healing. In addition, in this study, therapeutic ultrasound was used, which improved the healing of cutaneous grafts.

In more recent studies by Itakura *et al*.^22^, new effects related to the use of ultrasound were observed. According to Itakura *et al*.^22^, the most unobtrusive heat-up related to the use of therapeutic US promotes decreased inflammation and accelerated metabolic rate. Thus, moderate heating promotes increased blood flow, and may act in the treatment of chronic inflammation.

By the thermographic analysis of the images performed in this study and according to the literature^24^, the infrared emissions emitted by the animals are directly related to tissue perfusion and metabolism, with variations in the surface temperature changes in the circulation of the area. Thus, regions with higher metabolism presented higher temperatures captured by the camera and, consequently, hot spots detected and visualized through reddish/orange areas, when compared with regions of lower metabolic activity.

## Conclusion

The thermographic analysis of the images was effective in evaluating the healing process, being the use of thermography feasible to evaluate changes in the thermal standard in the surgical bed, besides the beneficial effects of the US.
